# Mucous Diarrhœa with Pregnancy

**Published:** 1873-05-15

**Authors:** F. K. Bailey

**Affiliations:** Knoxville, Tenn.


### MUCOUS DIARRHŒA WITH PREGNANCY.—DEATH AFTER CONFINEMENT.

BY F. K. BAILEY, M.D., KNOXVILLE, TENN.

December 4th, 1872—Mrs. L., aged about thirty-two; married six years; light complexion; feeble organization; much emaciated; and about eight months advanced.

Complains of pain in the abdominal and pelvic regions; has had diarrhœa for some time, attended with pain, and voids mucous discharges frequently during the twenty-four hours; tongue pale, with slight coat at the base; thirsty, with but little desire for food; pulse very soft and feeble—about 90 in a minute; has taken epsom salts, and no other medicine. Prescribed as follows:

℞.—Bromide potassii, ʒ ii.

Sulph. quinia, ℈ i.

Tinct. gentianæ comp., ℥ ss.

Tinct. cinch. comp., ℥ iss.—Mix.

F. mixtura sig. teaspoonful, four times daily. To take whisky-toddy freely, with rich beef soup. Also, to have an enema of fifteen drops tinct. opii every two hours till the diarrhœa is checked.

December 6th—Found patient much better; diarrhœa controlled completely, and pains relieved, which had been a source, of great annoyance.

The above course of treatment was followed up for a few days, and the symptoms were materially relieved.

I saw no more of the woman till the 17th inst., when I was called, and found her in labor. Arrived at the bedside at 10 A.M., and in an hour she was delivered of a male child, rather small in size, and showing no signs of life. The umbilical cord beat feebly, for a few minutes only. Placenta came away at once, and uterine contraction appeared sufficient.

The next morning she was as comfortable as could be expected, having slept about four hours during the night. There had been some irritability of the bowels, but no discharge. To take the mixture ordered at the first visit. To have soups freely, with milk-punch.

December 18th—Complains of pain and extreme soreness in the left iliac region; some fullness over the descending colon, with extreme tenderness on the slightest pressure; the lochial discharge scant, but not unnatural in color; no uterine pains; bowels moved once or twice, with the same appearance as at first; pulse very feeble and small, 124 in a minute; thirsty, but has some appetite; urine rather free; has slept a little. To continue stimulants and the bromide of potassii mixture.

December 19th — More feeble, but no change otherwise. To continue treatment.

December 20th, 9 A.M.—Had a severe chill about 10 last night, followed with heat of surface and increase of pulse; the lochial discharge has not been lessened, but rather increased, for the last two days; stomach not deranged. Gave sulph. quinia and pulv. Doveri, every three hours, with whisky and soup. 4 P.M.—Fever subsided, but abdominal soreness has increased; pulse very feeble and small—130 in a minute. To continue quinia.

December 21st, 9 A. M. — More feeble; bowels moved once during the night; discharges much as at former times; pulse 120, and *very* feeble. To continue powders, with stimulants. 5 P.M.—Failing; some hiccough; abdominal soreness somewhat abated; lochial discharge continues. To continue stimulants.

December 22d—Patient died at midnight.

I will remark that of late a few cases of puerperal fever have occurred in the city, and at about the same time embraced in the above history. It may be deemed probable that at the time of chill, in this case, a condition allied to that of puerperal peritonitis set in. The vital powers were so feeble that, if such was the case, no appreciable amount of reaction came up. The cases alluded to occurred in the hands of other medical men, and I have been unable to obtain a full history in any of them.

Two of the patients were young women about twenty, married in January, 1872; and both died on the same day. They were attended by different physicians, and the disease could not, therefore, have been conveyed from one to the other.

Another case was that of a lady, mother of a numerous family. I learned that the symptoms were characteristic of child-bed fever, and resulted favorably, but with considerable prostration.

Another still, was the mother of four or five children, and was not taken till the third or fourth day; result fatal. The two last cases were attended by Dr. Boyd, who also was the physician of one of the young women. The cases, however, were at considerable intervals, and consequently merely sporadic.

